# Flexible modelling of risk factors on the incidence of pneumonia in young children in South Africa using piece-wise exponential additive mixed modelling

**DOI:** 10.1186/s12874-020-01194-6

**Published:** 2021-01-11

**Authors:** Jordache Ramjith, Kit C.B. Roes, Heather J. Zar, Marianne A. Jonker

**Affiliations:** 1 Department for Health EvidenceBiostatistics Research GroupRadboud Institute for Health Sciences, Radboud University Medical Center, Nijmegen, Netherlands; 2 Department of Paediatrics and Child HealthRed Cross War Memorial Children’s Hospital and SA-MRC unit on Child and Adolescent Health, University of Cape Town, Cape Town, South Africa

**Keywords:** Cox model, Proportional hazards, Time-varying effects, Recurrent events, Piece-wise exponential model, Additive model, Survival analysis

## Abstract

**Introduction:**

Recurrent episodes of pneumonia are frequently modeled using extensions of the Cox proportional hazards model with the underlying assumption of time-constant relative risks measured by the hazard ratio. We aim to relax this assumption in a study on the effect of factors on the evolution of pneumonia incidence over time based on data from a South African birth cohort study, the Drakenstein child health study.

**Methods:**

We describe and apply two models: a time-constant and a time-varying relative effects model in a piece-wise exponential additive mixed model’s framework for recurrent events. A more complex model that fits in the same framework is applied to study the continuously measured seasonal effects.

**Results:**

We find that several risk factors (male sex, preterm birth, low birthweight, lower socioeconomic status, lower maternal education and maternal cigarette smoking) have strong relative effects that are persistent across time. When time-varying effects are allowed in the model, HIV exposure status (HIV exposed & uninfected versus HIV unexposed) shows a strong relative effect for younger children, but this effect weakens as children grow older, with a null effect reached from about 15 months. Weight-for-length at birth shows a time increasing relative effect. We also find that children born in the summer have a much higher risk of pneumonia in the 3-to-8-month age period compared with children born in winter.

**Conclusion:**

This work highlights the usefulness of flexible modelling tools in recurrent events models. It avoids stringent assumptions and allows estimation and visualization of absolute and relative risks over time of key factors associated with incidence of pneumonia in young children, providing new perspectives on the role of risk factors such HIV exposure.

**Supplementary Information:**

The online version contains supplementary material available at (10.1186/s12874-020-01194-6).

## Content

## Introduction

Children may experience multiple (recurrent) pneumonia episodes. The episodes within a child’s longitudinal profile may not be independent. Two common regression models in a recurrent events framework that can take into account this dependence are the Poisson regression model with an individual random effect (a mixed-effects model) and the shared frailty model [1] – an extension of the Cox proportional hazards (CPH) model [2]. In these models the individual random intercept describes the correlation between recurrent events for a single individual. The concept of shared random intercept in a multiple outcomes scenario is based on the idea that individuals may be heterogeneous but each individual’s risk of failure may be homogenous for the different events [1].

The estimated risk effects in these models are usually presented in terms of a measure for relative risks i.e. the incidence rate ratio for the Poisson regression model or the hazard ratio for the shared frailty model. The main assumption in these models are, that, conditional the random intercepts, these measures of relative risks are proportional over time. This is obviously not a problem if the effects are indeed time-constant, but flexibility in these models is needed otherwise.

Previous results [3] from the Drakenstein Child Health Study (DCHS) show several significant risk factors for the incidence of pneumonia in the first year of life based on a mixed-effects Poisson regression model in which the incidence rate ratios of risk factors are assumed to be constant over the first year of life. These results are useful and helpful in the understanding of relative risks averaged over a short time period. However, for longer follow-up periods, as in the analysis presented in this paper, it is biologically plausible that these ratios are not constant over time. As a consequence, important periods in which the relative risks are high or important changes of the relative risks happen over time may be missed. It may therefore be important to use a model that is flexible enough to detect these periods of high relative risks or changes, but is still sufficiently rigid to be able to interpret the results; models that are too rigid may mask these risk periods for instance by averaging over a longer period.

An accurate estimate of the absolute risk (underlying hazard), possibly changing over time, is as important as the relative risks in understanding the evolution of the risk over time. Namely, relative risks can be easily misunderstood; for instance a high relative risk may still be clinically irrelevant if the overall hazard rate is low at that particular point in time. Conditional on the random intercept, the mixed-effects Poisson regression model assumes a constant underlying hazard over time. This assumption may be too rigid, which makes the model unsuitable for our aim. In the CPH frailty model, parameters are estimated by maximizing the partial likelihood function where the baseline hazard is not specified and not explicitly estimated in survival analysis software. Because the baseline hazards are needed to estimate the absolute risks over time, this model is also less appropriate. To relax the constant hazard assumptions and to be able to estimate absolute risks in addition to relative risks, we propose to model the underlying hazard flexibly by using splines. To model the potential time-varying effect of the risk factors (discussed in the previous paragraph) splines are also used. To avoid overfitting of the data, a penalty for the number of model parameters is applied.

More specifically, a piece-wise exponential additive mixed model (PAMM) approach is used to model the effect of risk factors on the hazard of pneumonia in a recurrent events framework. PAMMs for time-to-event data is a combination of piece-wise exponential models (PEMs) [4] and generalized additive mixed models (GAMMs) [5], and was introduced by [4, 6] for modelling smooth non-linear baseline hazards, non-linear effects of covariates, time-varying hazard ratios in time-to-event models and more complex non-linear interactions. In this paper the PAMM is applied to model the effect of risk factors on the hazard of recurrent pneumonia.

### Pneumonia background

Globally, pneumonia is the single major cause of mortality in young children outside the neonatal period, with a disproportionately higher number of children dying from pneumonia in Africa [7–10]. The seriousness of pneumonia can range from mild to life threatening, more seriously affecting those who are immune compromised, malnourished or young children.

The introduction of vaccination programmes like pneumococcal conjugate vaccines (PCV) and *Haemophilus influenzae* type B immunization decreased the incidence and severity of childhood pneumonia [11–14]. However, even with high coverage for PCV13 (a PCV that protects against 13 serotypes of pneumococcus) the incidence of pneumonia remains high, particularly in the first six months of life (0.55 episodes per child year in the DCHS birth cohort [3, 14]).

Children living in fragile environments are especially at high risk for pneumonia and mortality. Knowing the effect of risk factors for pneumonia incidence is important for further understanding of pneumonia aetiology and for reducing childhood pneumonia incidence and mortality [13, 15]. Known risk factors for childhood pneumonia, subdivided into four categories, are [3, 10, 11, 13]:

- Environmental – poverty, pollution, crowding, cigarette smoke exposure, season,
- Maternal – low education, HIV, psychosocial distress,
- Child – nutrition, male sex, HIV, preterm birth, low birthweight, lack of breastfeeding, and
- Health system – lack of access to preventive treatment (immunization).

For a better understanding of pneumonia aetiology with respect to the effect of risk factors and improved organisation of medical care it is important to know

- in which periods certain subgroups are at high(er) risks, and
- how the relative risks possibly evolve over time.

Models to understand the effect of risk factors on pneumonia incidence with recurrences often assume that the relative risks between levels of a covariate are constant over time (conditional on the random individual intercept in recurrent events models). From a biological point of view, the developmental trajectory of babies and young children under two years is rapid, much more so than at older ages. It is thus more likely that the relative effect of covariates vary over time at young age. In this paper, we aimed to analyse data from the DCHS study using a model that allows a flexible smooth baseline hazard and flexible smooth time-varying relative risks, i.e. PAMM.

## Methodology

### The Drakenstein child health study data

The DCHS is a birth cohort designed to investigate the incidence and aetiology of childhood pneumonia after the introduction of the PCV13 and HiB vaccines, and the factors affecting the disease. The study is located in two sites, Mbekweni and TC Newman, in a low socio-economic area in Paarl, South Africa [14, 16]. In the DCHS 1137 children were enrolled, who were born between May 2012 and September 2015. Children were followed prospectively from birth with active surveillance for pneumonia [3]. Pneumonia was diagnosed according to the WHO clinical case definitions [17]. Repeated events were defined as any events that happened more than 14 days after a previous event. The episode dates were known exactly. Data of the first two years of life or until termination (death, end of study, lost to follow-up) was analyzed. Congenital cases were excluded from the analysis.

### Possible risk factors

In this paper, the possible risk factors studied are sex, HIV exposure, weight-for-length (WfL) at birth, prematurity, low birthweight, study site, socioeconomic status, crowding, maternal smoking, exposure to indoor air pollution, maternal education and seasonality. HIV exposure refers to a comparison between children who are HIV exposed and uninfected (HEU) and HIV unexposed (HU) children. The HIV exposed and uninfected children are exposed by means of being born to HIV positive mothers who are on antiretroviral therapy, but these children themselves are HIV negative. Low birthweight is quantified as having a birthweight under 2500 grams. Preterm birth is defined as having a gestational age less than 37 weeks. Weight-for-length (WfL) at birth Z scores are computed from the WHO algorithms [18]. Low WfL at birth is then indicated as a WfL Z score less than or equal to -2. The two study sites are TC Newman and Mbekweni. It must be noted that owing to the history of apartheid in South Africa, these study sites are culturally different with respect to ancestry and language. Socioeconomic status is measured based on a composite score of asset ownership, household income, employment and education, adapted from items used in the South African Stress and Health Study (SASH) [19]. A score below the median are referred to as lower socioeconomic status, while scores above the median are referred to as a higher socioeconomic status. Maternal smoking is derived from urine cotinine levels at an antenatal visit and categorised into exposed, where the mother actively smoked, or unexposed, where the mother did not actively smoke. Exposure to indoor air pollution, specifically fossil heating, is categorised as exposed (wood, gas, paraffin or coal used for heating in the household) and unexposed. Maternal education is categorised as lower (primary and some secondary schooling) and higher (at least completed secondary schooling). Crowding is represented by the number of other children under 5 years in the household where >1 child under 5 is representative of crowding. For seasonality, we model two covariates, season at birth and current season, of which both effects may vary over time. In both instances, we model seasonality as a continuous covariate. So, season at birth is recorded as the day of the year in which the child is born and current season as a time-varying covariate defined as the current day of year at risk.

### Statistical models

We describe the PAMMs approach tailored for modelling recurrent events in the DCHS data. We only applied univariable models with a single possible risk factor since the primary aim is to investigate association between the possible risk factors (mentioned before) and the incidence of pneumonia over time. We consider models with binary covariates and models with the continuously measured seasonal covariates. For the binary covariates, we consider two scenarios. In the first scenario we assume time-constant effects only, whereas in the second scenario we allow time-varying effects. These corresponding models are described by means of their hazard functions given below. For seasonality as a continuous covariate, we look at two similar models, described by their hazard functions also given below.

The hazard functions are given conditional on an individual frailty term *z*_*i*_. The frailty term is a random gaussian distributed term that is specific for each child (*i*) in the dataset. This frailty term is included to take into account that children may have multiple recurrent events in the dataset. For more details, see [5]. For describing the three models, the observation window from 0 to 2 years (728 days) is partitioned into *J* intervals with cutpoints $0={\tau }_{0}<{\tau }_{1}<\dots <{\tau }_{J}=728$ where the *j*^*th*^ interval is defined as (*τ*_*j*−1_,*τ*_*j*_], extending from and excluding the (*j*−1)^*st*^ boundary to and including the *j*^*th*^ boundary. The cutpoints ${\tau }_{1}<\dots <{\tau }_{J-1}$ are the ordered unique event times in the data.

### Binary covariates models

Suppose a covariate *x*_*i*_ for the *i*^*th*^ child has two levels which are denoted by 0 and 1 for simplicity. For the model with a time constant effect of the covariate, the hazard rate at time *t* *ε* (*τ*_*j*−1_,*τ*_*j*_], for the *i*^*th*^ child conditional on the covariate and frailty *z*_*i*_ equals

1 $$  \lambda \left(t|{z_{i},x}_{i}\right)={\text{exp} \left({\beta }_{0}+{\beta}_{1}x_{i}+f_{0}\left(t_{j}\right)+z_{i}\right)\ }  $$

and for the model with the time-varying effects the conditional hazard equals

2 $$  \lambda \left(t|{z_{i},x}_{i}\right)={\text{exp} \left({\beta }_{0}+{\beta }_{1}x_{i}+f_{0}\left(t_{j}\right)+x_{i}f_{1}\left(t_{j}\right)+z_{i}\right).\ }  $$

In both models *t*_*j*_ is a fixed time point across individuals within the *j*^*th*^ interval (*τ*_*j*−1_,*τ*_*j*_], usually defined as the end time of the interval or the midpoint. We chose the end time *t*_*j*_=*τ*_*j*_. The model formulation of the above models are expressed as conventional GAMMs. Model 1 is equivalent to the standard survival frailty model formulation *λ*(*t*|*z*_*i*_,*x*_*i*_)=*λ*_0_(*t*) exp(*β*_1_*x*_*i*_+*z*_*i*_) where the baseline hazard is specified as *λ*_0_(*t*)= exp(*β*_0_+*f*_0_(*t*_*j*_)). The *β*_0_ could be interpreted as the time-average log-hazard of children with the covariate *x*_*i*_ equal to zero (the first category) and *β*_1_ is the time-average difference between log-hazards for the second and the first category of the covariate. Further, *f*_0_ represents the smooth deviation from the average baseline log-hazard rate *β*_0_ over time, noting that *f*_0_ in the two models may differ notwithstanding the notation (this may also be true for *β*_0_ and *β*_1_). In model 2, *f*_1_ is a difference smooth function, which is the smooth shifts over time, for the difference between the effects for the second and first category of the covariate, from the average effect of the covariate *β*_1_ [20]. Further, it can be seen that the conditional hazard ratio for the covariate over time equals *HR*(*t*)=exp(*β*_1_+*f*_1_(*t*_*j*_)) ∀ *t* *ε* (*τ*_*j*−1_,*τ*_*j*_] for a child with *x*_*i*_=0 and one with *x*_*i*_=1, who have identical frailty values. The smooth functions, *f*_*k*_, k=0,1, evaluated at the point *t*_*j*_ are equal to a weighted sum of *S* simpler, fixed basis functions $b_{s,},\ s=1,\dots.S,$ in time *t*_*j*_, weighted by its corresponding regression coefficients *β*_*k*,*s*_, i.e. $f_{k}\left (t_{j}\right)={\sum \nolimits }^{S}_{s=1}{{\beta }_{k,s}}\times b_{s}\left (t_{j}\right),$ for k=0,1. For identifiability of the model parameters, the sum of each smooth function across time is set to equal 0, i.e. ${\sum \nolimits }_{j}{f_{k}\left (t_{j}\right)=0}$. The basis functions *b*_*s*_(*t*) are usually represented by splines, for which there are several options. For the analysis described in this paper, we use thin plate regression splines [21].

The individual frailties *z*_*i*_ account for the possible correlation between multiple episodes of pneumonia from the same child. The *zi*′s are assumed to be normally distributed with a mean of 0 and a constant variance *σ*^2^. The variables exp(*z*_*i*_) act to multiplicatively increase or decrease the hazard of a child over time, so that children with *z*_*i*_>0 have a hazard higher than the mean and children with *z*_*i*_<0 have a hazard lower than the mean. Children with *z*_*i*_=0 have the average hazard. We interpret the model parameters for an average child, for *z*_*i*_=0 (in the estimation of the parameters, the full likelihood is obtained by integrating out the frailty variables from the joint likelihood). Please note that this is not the same as leaving the frailty variables out of the model because the frailties affect the parameter estimates.

Model 2 generalizes model 1 into a larger model that makes no assumptions about the shape of hazard ratio over time, whereas model 1 assumes a time-constant hazard ratio. More specific, the hazard ratio for the two groups in model 1 is *HR*=exp(*β*_1_) and is constant over time, whereas in model 2 the time-varying hazard ratio is *HR*(*t*)=exp(*β*_1_+*f*_1_(*t*_*j*_)). The latter consists of two parts; *β*_1_, the average log hazard ratio and *f*_1_(*t*_*j*_), the time-varying component of the log hazard ratio. If the function *f*_1_ is constant and equal to zero the two models are equivalent.

### Seasonality models

We model both season at birth and season during follow-up (i.e. current season). We model season at birth, defined by day of the year the child was born (*x*_1_), as a time-varying effect. We model the current season at risk as a time-varying covariate, which we define *x*_2_(*t*) as the day of year for the start date of the interval. We use the start of the interval because this is the start season of risk within the interval. For simplicity of notation, we write *x*_2_ instead of *x*_2_(*t*) henceforth. So, in the model we assume that season is constant within the interval but varies between intervals and that the current season effect is periodic, in the sense that it equals the effect one year later. For the two scenarios just described, the hazard rate at time *t*, for the *i*^*th*^ child conditional on the individual random effect *z*_*i*_ and either *x*_1*i*_ or *x*_2*i*_ the seasonal covariates as described before, is given by

3 $$  \lambda \left(t|{z_{i},x}_{k,i}\right)={\text{exp} \left({\beta }_{0}+g_{k}\left(t_{j},x_{k,i}\right)+z_{i}\right)\ },  $$

for *t* *ε* (*τ*_*j*−1_,*τ*_*j*_], *t*_*j*_=*τ*_*j*_ and *k*=1,2. The interaction function *g*_*k*_(*t*_*j*_,*x*_*k*,*i*_) models the interaction of continuous season at birth if *k*=1, or current season if *k*=2, and age (time) and allows the effect season on the hazard to be smooth and non-linear at each point in time, and also allows the effect each season (day of year) to be smoothly and non-linearly time-varying. This smooth function refers to a tensor product represented by *S*×*M* basis functions such that $g_{k}\left (t_{j},x_{k,i}\right)={\sum \nolimits }^{S}_{s=1}{{\sum \nolimits }^{M}_{m=1}{{\alpha }_{k,m,s}}}\times b_{s}\left (t_{j}\right)\times b_{m}\left (x_{k,i}\right)$ for *k*=1,2. In these models, we use cubic regression spline basis functions for modelling the time effect and a cyclic cubic regression spline basis function for modelling seasonality effect [22]. A cyclic cubic regression spline is a penalized cubic regression spline whose ends match up to a second derivative. We use the cyclic cubic regression spline basis to ensure continuity in the hazard from day 365 to day 1. The sum-to-zero constraints are applied to all smooth functions for identifiability.

The three different models with hazard functions in (1) – (3) are referred to as models 1 to 3, where the number corresponds to the number of the corresponding expression of the hazard function.

### Estimation

The likelihood function for the PAMM is equivalent to the full likelihood of the CPH model if the baseline hazards were assumed constant within the intervals. It has also been shown that the likelihood of the piece-wise exponential model is proportional to the likelihood of the Poisson model including an offset [23]. The offset for the PAMM is the log of the amount of actual time an individual spends in an interval. This allows the model to account for an individual’s exact event times, making the model a model for continuous time-to-event data [4]. For a recurrent events PAMM, the likelihood also includes the subject-specific frailty. Penalized negative log likelihood functions are derived in which the model (negative log) likelihood is modified by the addition of a penalty for each smooth function, penalizing its ‘wiggliness’. We estimate the unknown parameters in the model with the fast restricted maximum likelihood method, where numerical maximization of the likelihood is performed with Penalized Iteratively Re-weighted Least Squares (P-IRLS) [5, 24]. We use the R packages pammtools and mgcv for the data restructuring and analysis [25, 26]. A guide to performing the analysis, with R code, is provided as a Supplementary file.

### Statistical analysis and model selection

The effects of the smooth functions *f*_*k*_(*t*_*j*_) (*k*=0,1) can be expressed in terms of the estimated degrees of freedom (EDF) and a corresponding *p*-value [27, 28]. This EDF, is not like degrees of freedom that are usually used, but rather it is more like the degree of the polynomial needed to describe curvature. It provides an idea for how “wiggly” the best fitted smooth function is over time (best fitted in terms of the maximal penalized likelihood function). The *p*-values are the result of testing the null hypothesis of a zero effect of the indicated smooth function, i.e. whether *f*_*k*_(*t*_*j*_)=0, *k*=0,1 for $j=1\dots J$. A high EDF implies a more complex shape (or ”wiggliness”) of the penalized smooth function. Note that if the *p*-value is below the pre-specified significance threshold we may conclude that the effect is time varying and model 2 is preferred over model 1, whereas if the *p*-value is above the threshold this cannot be concluded and model 1 is chosen. We refer to the chosen model for each covariate as the final model. It is important to note that making this choice based on a *p*-value threshold is one option to decide between models, but not the only one.

In this paper, we show the results for the estimated difference smooth function ${\hat {f}}_{1}\left (t_{j}\right)$ and not ${\hat {f}}_{0}\left (t_{j}\right)$ since ${\hat {f}}_{1}\left (t_{j}\right)$ is used to indicate the time-varying effect and thus allows us to choose between model 1 and 2 for each covariate. An EDF=1 implies that the penalized smooth function, and thus the estimated time-varying log hazard ratio, ${\text {log} \widehat {HR}(t)\ }={\widehat {\beta }}_{1}+{\hat {f}}_{1}\left (t_{j}\right)$, is estimated to be linear over time. Note that high EDF doesn’t mean greater significance. These effects are better understood visually, illustrated in the “Results” section. 95% confidence intervals for the results are calculated from the linear predictor at the respective intervals.

The results for seasonality are only expressed visually as heatmap plots representing a 3-dimensional association between seasonality, time (age) and the hazard or hazard ratios.

Since PAMMs assume constant hazard rates within intervals, the hazard rate and the daily incidence rate (the number of episodes per child day) are equal within intervals. To allow for a better epidemiological interpretation, both are multiplied by 365.25 to obtain a yearly incidence rate within each interval (i.e. the number of episodes per child year), as well as a “yearly hazard rate”.

## Results

### Data description

The sample consists of 1137 children, of whom 445 (39%) experienced ≥1 pneumonia episode. Of these 445 children, 236 (53%) had only one episode while a further 209 (47%; 18% of the entire sample) children experienced at least a second episode. In Table 1 we show a cross-tabulation of how the different risk factors are distributed across the study site. From the total of 1137 children, approximately 628 (55%) of children were from Mbekweni and 509 (45%) from TC Newman. The largest differences across study sites were HIV exposure (228 (36%) exposed in Mbekweni and 16 (3%) exposed in TC Newman), socioeconomic status (356 (57%) with lower SES in Mbekweni and 211 (42%) with lower SES in TC Newman), cigarette smoke exposure (91 (14%) in Mbekweni and 261 (51.3%) in TC Newman) and exposure to indoor air pollution (132 (21%) in Mbekweni and 7 (1%) in TC Newman).

**Table 1:** Frequency distribution of risk factors across the sites

Risk factors	Mbekweni n (%)	TC Newman n (%)
Total	628 (55.2)	509 (44.8)
HU	397 (63.2)	493 (96.9)
HEU	228 (36.3)	16 (3.1)
Girls	316 (50.3)	232 (45.6)
Boys	312 (49.7)	277 (54.4)
Not low WfL	386 (61.5)	299 (58.7)
Low WfL	174 (27.7)	154 (30.3)
Not preterm birth	522 (83.1)	425 (83.5)
Preterm birth	106 (16.9)	84 (16.5)
Not low birthweight	554 (88.2)	414 (81.3)
Low birthweight	74 (11.8)	95 (18.7)
Lower SES	356 (56.7)	211 (41.5)
Higher SES	272 (43.3)	298 (58.5)
Uncrowded	487 (77.5)	344 (67.6)
Crowded	141 (22.5)	165 (32.4)
No maternal smoking	504 (80.2)	232 (45.6)
Maternal smoking	91 (14.4)	261 (51.3)
Unexposed to Indoor air pollution	311 (49.5)	387 (76.0)
Exposed to indoor air pollution	132 (21.0)	7 (1.4)
High maternal education	239 (38.1)	206 (40.5)
Low maternal education	389 (61.9)	303 (59.5)

### The estimated baseline hazard and baseline cumulative incidence proportion

**Fig. 1:**
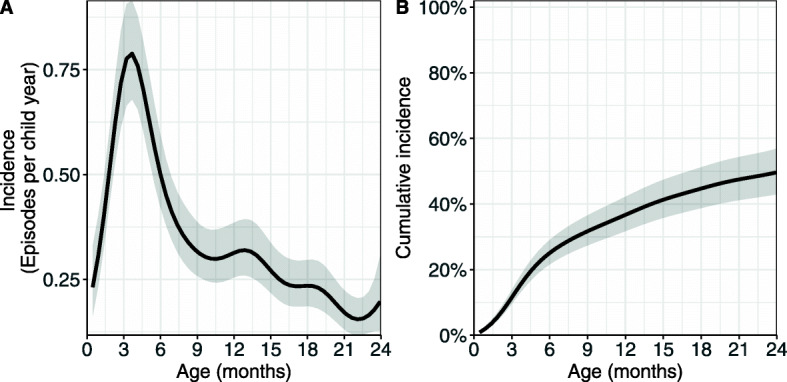
**a** The estimated baseline incidence rate and **b** estimated baseline cumulative incidence probabilities for the first episode and recurrent episodes over time, with 95% confidence intervals (shaded area)

The estimated average hazard rate in the first two years of life for the reference and comparison groups i.e. $\widehat {\lambda }={\text {exp} \left ({\widehat {\beta }}_{0}\right)\ }$ and $\widehat {\lambda }={\text {exp} \left ({\widehat {\beta }}_{0}+{\widehat {\beta }}_{1}\right)\ }$ have also been rescaled to an annual rate in Table 2 such that the estimated average incidence rate (episodes per child year) in the first two years of life $\left (\overline {IR}\right)\ $ for the reference and comparisons group are ${\mathrm {365.25\times exp} \left ({\widehat {\beta }}_{0}\right)\ }$ and ${\mathrm {365.25\times exp} \left ({\widehat {\beta }}_{0}+{\widehat {\beta }}_{1}\right)\ }$ respectively. Estimates of the incidence rates as the number of episodes per child year and the proportion of children accumulated over time who have experienced at least one episode of pneumonia are presented in Fig. 1.

**Table 2:** Estimated average incidence rate $\left (\overline {IR}\right)$ in number of episodes per child year and estimated hazard ratios for the different covariates from model 1 i.e. $\widehat {HR}=\text {exp} \left ({\widehat {\beta }}_{1}\right)$, and for model 2 the estimated average incidence rate, $\left (\widehat {\overline {HR(t)}}\right)={\text {exp} {\widehat {\beta }}_{1}\ }$, as well as the estimated degrees of freedom (EDF) for the time-varying smooth function in the hazard ratio, ${\hat {f}}_{1}(t)$

	Model 1		Model 2		
	$\overline {IR}$ (95% CI)	$\widehat {HR}\ $ (95% CI; *p*-value)	$\overline {IR}$ (95% CI)	$\widehat {\overline {HR(t)}}$ (95% CI; *p*-value)	EDF (*p*-value)
HU	0.29 (0.26, 0.32)		0.29 (0.26, 0.33)		
HEU	0.42 (0.35, 0.51)	1.49 (1.21, 1.83; *p*<0.001)	0.40 (0.33, 0.48)	1.37 (1.10, 1.71; *p*=0.004)	1.00 (*p*=0.036)
Girls	0.25 (0.22, 0.29)		0.25 (0.22, 0.29)		
Boys	0.38 (0.34, 0.43)	1.53 (1.28, 1.84; *p*<0.001)	0.37 (0.33, 0.43)	1.48 (1.22, 1.79; *p*<0.001)	1.00 (*p*=0.261)
Not low WfL	0.30 (0.26, 0.33)		0.29 (0.25, 0.33)		
Low WfL	0.29 (0.25, 0.35)	0.99 (0.81, 1.22; *p*=0.926)	0.31 (0.26, 0.37)	1.07 (0.87, 1.33; *p*=0.522)	1.00 (*p*=0.023)
Mbekweni	0.34 (0.30, 0.38)		0.33 (0.29, 0.38)		
TC Newman	0.29 (0.25, 0.33)	0.85 (0.71, 1.02; *p*=0.074)	0.29 (0.25, 0.34)	0.89 (0.73, 1.07; *p*=0.212)	1.00 (*p*=0.162)
Not preterm birth	0.30 (0.27, 0.33)		0.29 (0.26, 0.33)		
Preterm birth	0.42 (0.34, 0.51)	1.41 (1.12, 1.77; *p*=0.003)	0.42 (0.34, 0.52)	1.43 (1.12, 1.81; *p*=0.003)	1.00 (*p*=0.752)
Not low birthweight	0.29 (0.26, 0.32)		0.29 (0.26, 0.32)		
Low birthweight	0.46 (0.37, 0.56)	1.56 (1.23, 1.97; *p*<0.001)	0.47 (0.37, 0.58)	1.61 (1.26, 2.05; *p*<0.001)	2.11 (*p*=0.168)
Lower SES	0.37 (0.32, 0.41)		0.37 (0.33, 0.42)		
Higher SES	0.26 (0.23, 0.30)	0.72 (0.60, 0.87; *p*<0.001)	0.26 (0.22, 0.30)	0.69 (0.57, 0.83; *p*<0.001)	1.00 (*p*=0.127)
Uncrowded	0.30 (0.27, 0.34)		0.30 (0.27, 0.34)		
Crowded	0.34 (0.29, 0.41)	1.14 (0.94, 1.39; *p*=0.177)	0.35 (0.29, 0.41)	1.16 (0.95, 1.43; *p*=0.152)	1.01 (*p*=0.635)
No maternal smoking	0.28 (0.25, 0.32)		0.28 (0.25, 0.32)		
Maternal smoking	0.38 (0.33, 0.45)	1.35 (1.12, 1.64; *p*=0.002)	0.39 (0.33, 0.45)	1.38 (1.13, 1.68; *p*=0.001)	1.00 (*p*=0.537)
Indoor air pollution unexposed	0.32 (0.28, 0.36)		0.32 (0.28, 0.36)		
Indoor air pollution exposed	0.27 (0.21, 0.36)	0.85 (0.63, 1.15; *p*=0.279)	0.26 (0.20, 0.35)	0.83 (0.61, 1.12; *p*=0.224)	2.00 (*p*=0.337)
High maternal education	0.27 (0.23, 0.32)		0.28 (0.24, 0.33)		
Low maternal education	0.34 (0.30, 0.38)	1.24 (1.03, 1.50; *p*=0.022)	0.33 (0.30, 0.38)	1.18 (0.97, 1.44; *p*=0.093)	1.47 (*p*=0.218)

We ran a model without any covariates and estimated baseline hazard rates and cumulative incidence. The baseline hazard rates estimated in the model without covariates show that pneumonia incidence peaks when children are between three and four months and thereafter slowly decreases substantially, but this decrease slows down after children turn 9 months (Fig. 1a). From Fig. 1b, we see that approximately 25% of the children have a first episode of pneumonia by 6 months old and another 25% of the children will have a first episode by 2 years old. The average estimated incidence for the first two years of life is 0.31 (95% CI: 0.28, 0.35) episodes per child year.

### Analysis of binary risk factors

Model 1 represented by (1) is the model assuming time-constant effects of the covariates over time and model 2 represented by (2) is the model assuming flexible time-varying effects of the covariates over time.

**Fig. 2:**
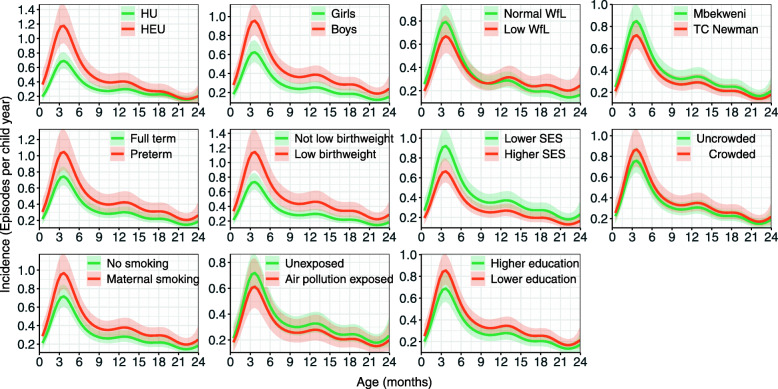
The incidence rates (hazard rates) for pneumonia from birth until 2 years by several risk factors estimated by univariable piece-wise exponential additive mixed models. For each variable we chose the time-constant model except for HIV exposure status and weight-for-length at birth where we had sufficient evidence for a time-varying effect. The shaded areas indicate the 95% confidence intervals

Estimates of the incidence rates as number of episodes per child year are presented in Fig. 2. Plots of the estimated cumulative incidence over time are in Fig. 5; this shows the proportion of children accumulated over time who have experienced at least one episode of pneumonia. In all figures, the time *t*_*j*_ on the x-axis is representative of all *t* *ε* (*τ*_*j*−1_,*τ*_*j*_].

### Relative risks (i.e. hazard ratios) over time

From the estimated hazards ratios in model 1 given in Table 2, we see that HEU, male sex, preterm birth, low birthweight, lower socioeconomic status, lower maternal education and maternal cigarette smoke exposure are significantly associated with relatively higher incidences of pneumonia over time for the first two years of life (relative to their counterpart categories).

Male sex, preterm birth, low birthweight, lower socioeconomic status, lower maternal education and maternal cigarette exposure do not have significant time-varying effects (model 2, Table 2) but children in each of these exposure categories have at least a 20% higher incidence averaged across time relative to children in the counterpart categories (HR >1.2; model 1, Table 2).

**Fig. 3:**
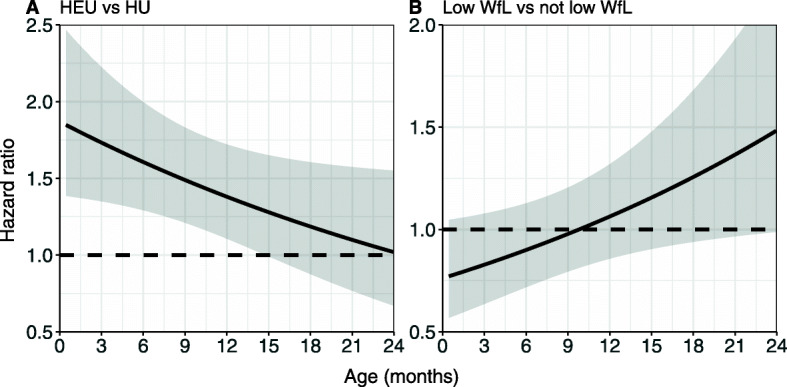
Time-varying hazard ratios over time $HR(t)={\text {exp}\ {\left (\widehat {\beta }_{1}+{\hat {f}}_{1}(t)\right)} \ \ }$for **a** HIV exposed and uninfected children versus HIV unexposed children, and **b** children with low weight-for-length Z-score at birth versus children who don’t have low weight-for-length Z-score at birth all with 95% confidence intervals (shaded area)

The time-varying effects smooth function for HIV exposure and weight-for-length at birth are statistically significant (*p*=0.036 and *p*=0.023 respectively) with EDF=1 implying a linear time-varying effect of the log hazard ratio (model 2, Table 2). The time-varying hazard ratios for both these factors (defined in “Statistical models” section) are visualized in Fig. 3, where we can see that the effect of HIV exposure is decreasing over time until it is approximately a null effect (*HR*=1) from around 15 months. Although the HR of weight-for-length at birth is not statistically significant in model 1 (Table 2), from model 2 the HR appears to start slightly protective after birth and then increases and appears increasingly harmful after approximately 9 months of age (Fig. 3).

Further, from the results of model 2 presented in Table 2, we also see the estimated averaged time-varying hazards ratios for all variables. These hazard ratios are similar to the estimated time-constant hazard ratios estimated from model 1, with the exception of HIV exposure, weight-for-length at birth and cigarette exposure. The estimated averaged time-varying hazard ratio for HIV exposure is lower in model 2 but still significant (p=0.004), while for weight-for-length at birth it is higher but still not significant (p=0.522).

**Fig. 4:**
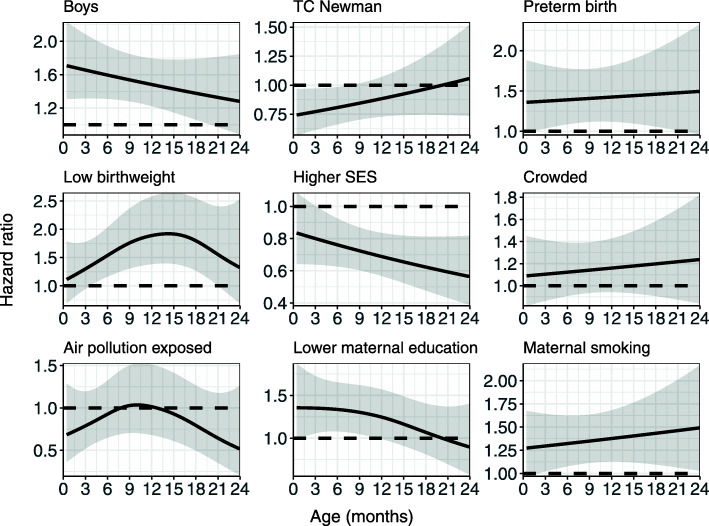
Time-varying hazard ratios over time $HR(t)={\text {exp}\ {\left (\widehat {\beta }_{1}+{\hat {f}}_{1}(t)\right)}}$ for the variables without significant evidence for time-varying trends, with 95% confidence intervals (shaded area)

The EDF for birthweight is 2.11 (Table 2) and is higher than that for all the other covariates but is statistically not significant (p=0.168). From the effects plots (Fig. 4), we see that the estimated HR is “wigglier” but a flat line easily fits within the 95% confidence intervals of the estimated curve. So, the time-varying HR estimated for birthweight in model 2 cannot be distinguished from a time constant HR. A similar interpretation can be given to all non-significant time-varying effects; whose hazard ratios are also visualized in Fig. 4.

**Fig. 5:**
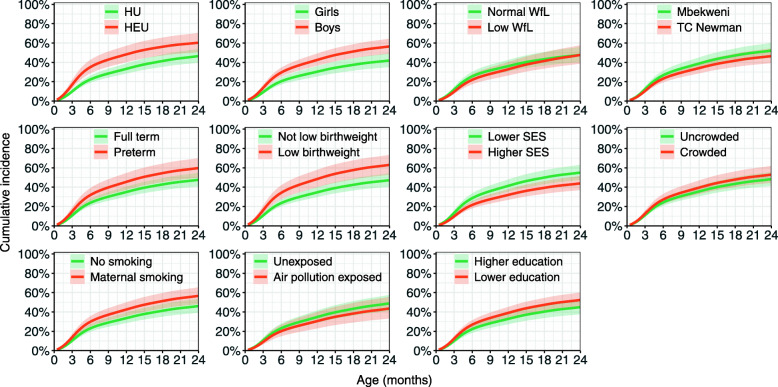
Cumulative incidence of pneumonia over the age of children under 2 years by several risk factors estimated by univariable piece-wise exponential additive mixed models. For each variable we chose the time-constant model except for HIV exposure status and weight-for-length at birth where we had sufficient evidence for a time-varying effect. The shaded areas indicate the 95% confidence intervals

### Estimated incidence over time

The estimated incidence curves over time from the final model for each covariate can be seen in Fig. 2 from birth until children are two years. Here we have used the final model (from model 1 and model 2) to estimate the hazard rate over time and then rescaled the hazard rate to correspond with incidence as number of episodes per child year (as explained at the beginning of this section). The shape, similar to the baseline, shows how covariates act to increase or decrease the hazards over time. For covariate effects estimated from model 1, this relative increase/decrease is constant over time, but not for model 2: all hazards, with the exception of HIV exposure status and WfL at birth, are modelled with model 1, which is clear from the figure, since we can see constant relative risks across time but for the three covariate with time-varying effects we see intersections in the hazard curves over time. It is interesting to note that all risk factors appear most important in the first six months. The risk factor associated with the largest absolute difference in incidence rates soon after birth is HIV exposure status (0.17 episodes per child year). HIV exposure and low birthweight, show the largest absolute differences between subgroups at the peak (around 3.5 months) i.e. approximately 0.5 episodes per child year.

Average incidences over the first two years of life are given from both models in Table 2. The highest estimated average incidences are for children with low birthweight, children who were born preterm and HEU children (more than 0.4 episodes per child year).

### Estimated cumulative incidence over time

The cumulative incidence over time from the final model for each covariate can be seen in Fig. 5. The largest cumulative incidence differences between subgroups after two years follow -up can be seen by sex and low birthweight. The estimated proportion of boys who have had an episode of pneumonia by age two years is approximately 0.15 more than the proportion for girls (approximately 0.55 for boys and 0.40 for girls). Likewise, the proportion of pneumonia in children born with low birthweight by two years of age is approximately 0.15 more than the proportion for children who were not born with low birthweight (0.60 versus 0.45).

### Analysis of seasonal effects

Before we describe the results of the analysis, it is important to define the day of year in South Africa as traditional seasons to better aid interpretation. The calendar dates for the seasons are as follows:

- Autumn/Fall – 1 March to 31 May (day 60 – day 151)
- Winter – 1 June to 31 August (day 152 – day 243)
- Spring – 1 September to 30 November (day 244 – day 334)
- Summer – 1 December to 28/29 February (day 335 – day 365; day 1 – day 59)

**Fig. 6:**
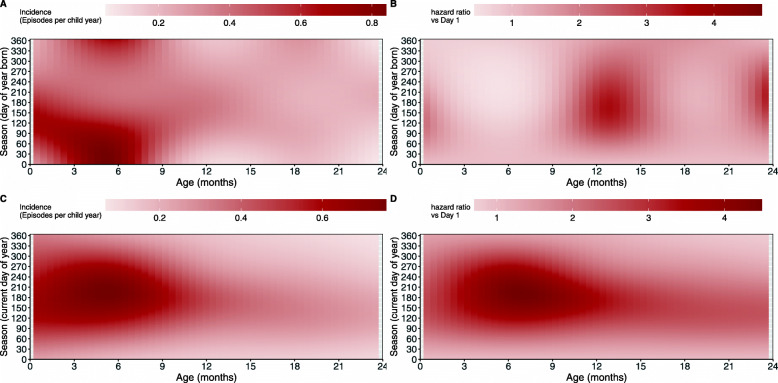
**a** Estimated incidence (episodes per child year) and **b** hazard ratios (relative to children born on the first day of the year, in mid-summer) over the age of children under 2 years from the season of birth and **c** estimated incidence (episodes per child year) and **d** hazard ratios (relative to children currently in the first day of the year, in mid-summer) over the age of children under 2 years at the current season

Figure 6 shows heatmap plots of the incidence of pneumonia across seasons and the age of the child estimated from model 3, as well as the hazard ratios for the hazards from all seasons (day of year the child is born or day of year at risk) relative to the hazards for children in the first day of the year for both seasonality variables.

In Fig. 6a, we can see that children born in the summer period have the highest peaks, at ages between 3 and 8 months. This peak is higher than for children born in any other period of the year. This is likely explained by a combination of factors: children’s natural peak of developing pneumonia is at around 3-4 months (as seen in the baseline incidence in Fig. 1) which is in the early winter period for children born in summer, a period in which the risk to develop pneumonia is increased in the whole population. These children also appear to have the lowest incidences when they are at their next summer at around 1 years of age. We also see that children born in the autumn days and early winter days have higher immediate incidence but their incidence slowly decreases as they grow older. Children born between the later winter days and spring appear to have more constant incidence even as they reach the summer days 6 months later.

In Fig. 6b, we compare the incidences for children born at all days of the year with children born on the first day of the year (so children who were born close to mid-summer in South Africa). We see that children born after the summer months but before mid-winter have a higher relative hazard for the first three months of life. Children born outside the summer days appear to have lower relative hazards between the critical ages of 3 to 6 months. However, these children tend to have much higher relative hazards when they are around 1 year old.

Figure 6c and d are interpreted slightly differently since the season is the current day of the year. The x-axis shows the age of the child while the y-axis is the current season so that every x-y combination in the plot represents the effects for a different group of children. In Fig. 6c, we see that children who are under 9 months old between early autumn and early spring have the highest incidence rates. We still see slightly darker shading between mid-autumn until the end of winter for children older than 9 months, indicating higher incidences compared to children at these ages outside of this seasonal period. This is better seen in the hazard ratio plot in Fig. 6d. The figure shows the ratios of the hazard for each current season (day of the year) relative to the hazard for children born on the first day of the year, for children of every age under 2 years. In this plot we see that children whose current season is from mid-autumn to the end of winter have higher hazards than children in peak summer, particularly for children between 3 to 18 months.

## Discussion

In this study we used piece-wise exponential additive mixed effects models to analyse univariable effects of covariates that were assumed either (1) time constant or (2) time-varying. We also looked at a more complex association between continuously measured seasonality and pneumonia incidence. The PAMMs approach has advantages in that it allows for various possibilities of smooth associations including non-linear effects of covariates, and non-linear time-varying effects. Since the model is fully parametric, it can be used in prediction models, and overfitting is avoided through penalization. Model selection of time-varying effects and possible non-linear effects of covariates are however limited to *p*-value based criteria and visualization of the effects, which may be difficult for researchers with less experience with flexible models to gauge on their own. An advantage of PAMMs is that the hazard rates can be directly translated into incidence rates as in Poisson regression models, allowing for better interpretation by clinical researchers compared to conventional hazard rates.

Through this analysis, we found that strong effects of some risk factors, specifically sex, low birthweight, preterm birth, low socioeconomic status, low levels of maternal education and maternal cigarette smoke exposure that persisted throughout the first 2 years of life. These are well known risk factors for the incidence of pneumonia [3, 10, 11, 13]. Further, we found that the relative risk for HIV exposure status is much higher soon after birth and decreases to a null effect as the child approaches an age of two years (with the lower limit of the confidence interval at 15 months). There are limited longitudinal studies that have compared the incidence of pneumonia in HEU and HU children. Slogrove et al. [29] performed a systematic review on studies that compare HEU and HU children, with respect to morbidity and mortality. One of their findings was that the greatest relative difference between HEU and HU infants in morbidity occurs beyond the neonatal period, during mid-infancy, having waned by the second year of life. This is consistent with our findings for pneumonia incidence. There has been evidence that HIV exposure status is strongly associated with pneumonia severity in the first 6 months of life [3, 30]. All pneumonia episodes in children under 2 months are classified as severe [17]. If all episodes under 2 months are severe and HIV exposure is linked to pneumonia severity, then this could be explaining why we see the relative risk of HIV decline over time. Further modelling is needed to investigate this.

We also found strong evidence of seasonal effects on the incidence of pneumonia, where we considered season of birth as a time-varying effect over the child’s age and also current season as a time-varying covariate over the first two years of life. The results suggest that incidence rates are higher in the winter season than in the summer season, especially within the first six months of life. Rudan et al. [10] and Janet et al. [31] highlight that the peak incidence of respiratory syncytial virus occurs for a period of 2 – 4 months during the cold seasons. Respiratory syncytial virus is the leading viral cause of hospitalized pneumonia in children who are immunised with PCV and HiB [32]. Further research is needed to study this association in the DCHS birth cohort.

We show that even though statistically insignificant in the analysis, WfL at birth has increasing relative risks over time in the first two years of life. A limitation in these findings is that this variable is a time-varying covariate, and should be modelled as such. However, the data for WfL as a time-varying covariate was limited.

Exclusive breastfeeding, although an important risk factor, was not included in the analysis because it is a special time-varying covariate where we can hypothesize that there are lagged effects of exposure on the hazard, and the effects of exposure accumulate over time. A different modelling approach [33] should be used for studying these time-varying covariates. This approach involves the flexible modelling of complex exposure-lag-response associations in time-to-event data, where multiple past exposures within a defined time window are cumulatively associated with the hazard. The model has not been extended to recurrent events, and is a consideration for future research.

Further research, highlighted by this work, is to explore the possible time-varying effect of HIV exposure status across pneumonia severity while accounting for important confounders, and to explore the associations between seasonality, pneumonia incidence and the presence of respiratory syncytial virus.

## Conclusion

It avoids stringent assumptions and allows estimation and visualization of relative risks over time of key factors associated with incidence of pneumonia in young children, providing new perspectives on the role of risk factors such HIV exposure.

Flexible modelling of the incidence of infectious diseases is needed for a better understanding of disease aetiology. This work highlights the usefulness of flexible modelling tools in recurrent events models. In particular, PAMMs extended for recurrent events, allowed for the flexible modelling of the effect of risk factors on pneumonia incidence over time with and without the stringent assumption of proportional hazards. In this study, we have shown that the relative risks of HIV exposure status, weight-for-length at birth and season at birth on pneumonia incidence varies with time. We have also shown complex non-linear effects of continuously measured seasonality. This type of flexible modelling can provide new perspectives on the role of risk factors on time to event outcomes. These perspectives need to be investigated further in future analysis.

## Supplementary Information

**Additional file 1** Supplementary file.

## Data Availability

Collaborations for the analysis of data are welcome. The DCHS has a large and active group of investigators and postgraduate students, and many have successfully partnered with researchers from other institutions. In particular, we encourage collaborations that lead to skills transfer and capacity building for postgraduate students. Researchers who are interested in datasets or collaborations can contact the PI, Heather Zar (heather.zar@uct.ac.za) with a concept note outlining the request. More information can be found on our website (http://www.paediatrics.uct.ac.za/scah/dclhs). An example dataset is included as a Supplementary file as *d**a**t**e**s**m**a**t*.*x**l**s**x*. This is a random sample of 200 children from the data. The children are anonymized and random id’s were made.

## Abbreviations

CPH: Cox proportional hazards
DCHS: Drakenstein child health study
EDF: Estimated degrees of freedom
GAMM: Generalized additive mixed models
HEU: HIV exposed and uninfected
HR: Hazard ratio
HU: HIV unexposed
PCV: Pneumococcal conjugate vaccines
PAMM: Piece-wise exponential additive mixed model
PEM: Piece-wise exponential models
P-IRLS: Penalized iteratively reweighted least squares
SASH: South African stress and health study
WfL: Weight-for-length
WHO: World health organization
